# Supplementary material for: Structural basis for recognition of diverse localizing mRNAs by Egl–BicD
Source: Nat Struct Mol Biol. 2026 May 5;33(5):882–93. doi: 10.1038/s41594-026-01794-8 (PMC13186709; doi:10.1038/s41594-026-01794-8)
Supplement: Supplementary file 1 — Supplementary Figs. 1–11, Tables 1–3 and References. [file 41594_2026_1794_MOESM1_ESM.pdf]

nature structural & molecular biology

Supplementary information https://doi.org/10.1038/s41594-026-01794-8

Structural basis for recognition of diverse localizing mRNAs by Egl–BicD

In the format provided by the authors and unedited

This Supplementary Information PDF file contains:

Supplementary Figs. 1–11
Supplementary Tables 1–3
Supplementary References

Supplementary Figures

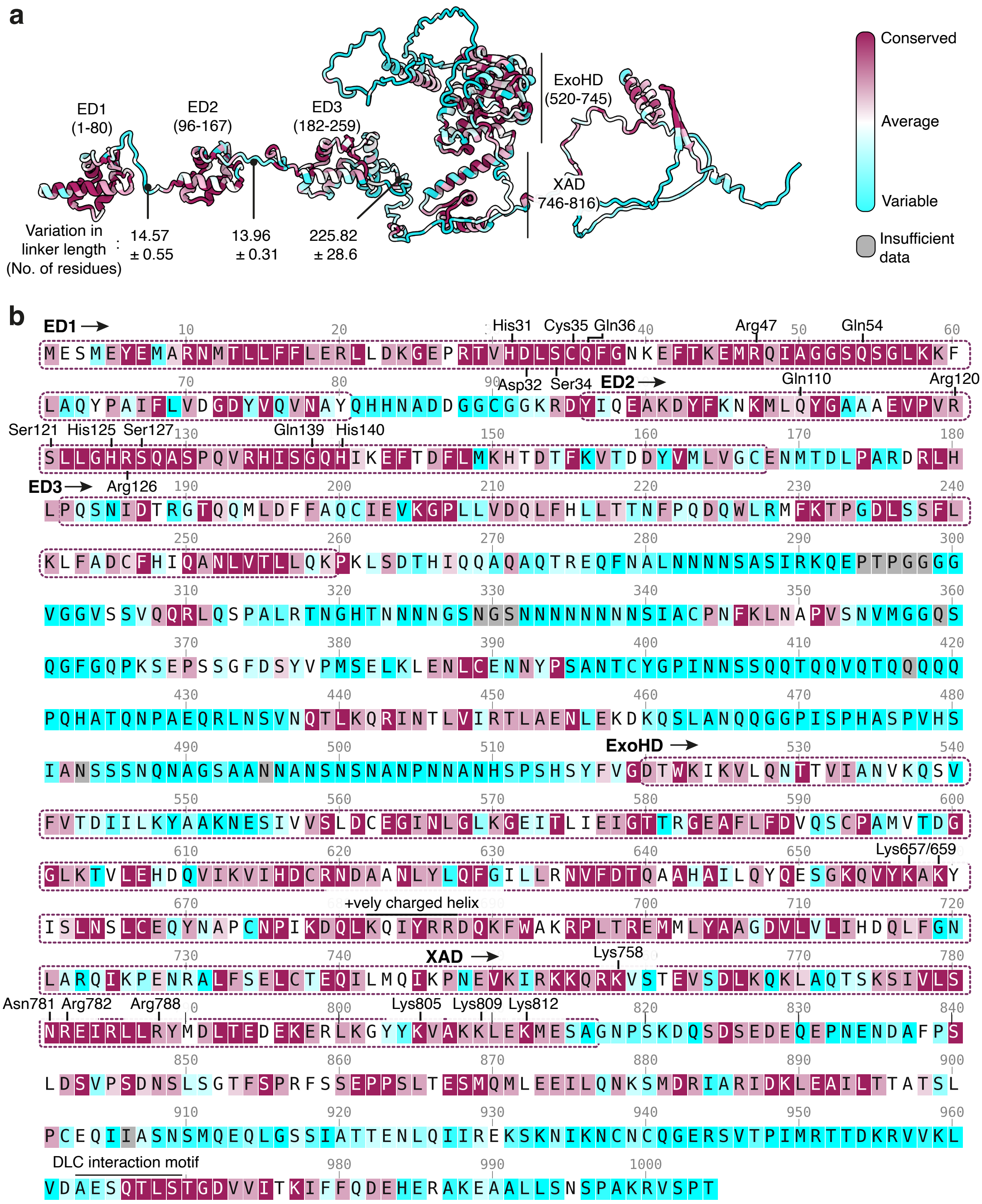

Supplementary Fig. 1 | Sequence conservation of Egl. a, Alphafold2 model of Egl shown in Fig. 1b colored by sequence conservation across 108 Egl orthologues. The different domains of Egl are indicated. Interdomain linker regions display low sequence conservation but relatively consistent lengths (particularly those between the EDs), as shown by the mean number of residues ± s.d. for each linker. **b**, Sequence of Egl annotated with domain boundaries and colored by residue conservation using the same scale as in **a**. Residues implicated in RNA recognition are denoted above the sequence. DLC, dynein light chain.

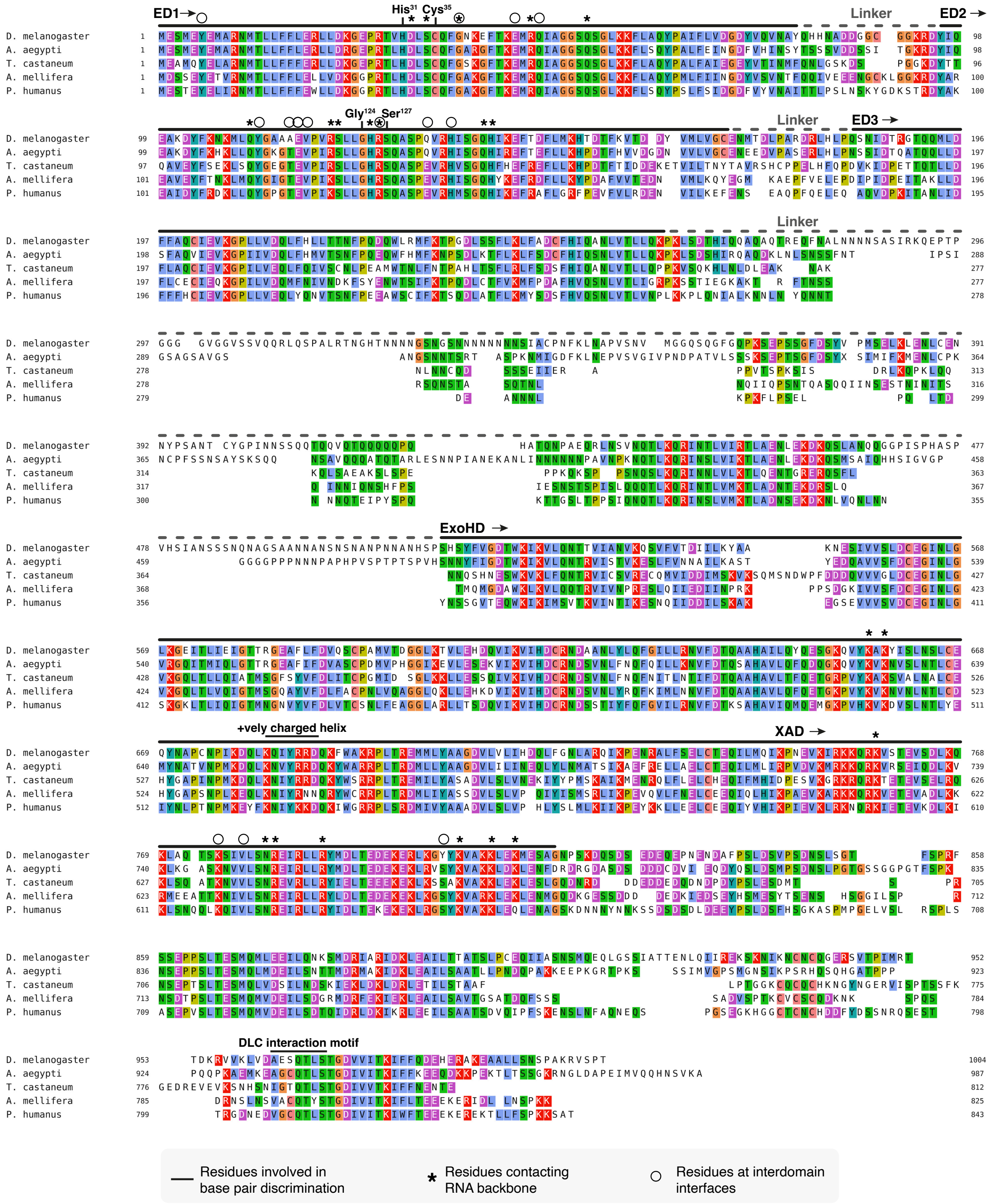

Supplementary Fig. 2 | Multiple sequence alignment of Egl orthologues across representative insect species. Amino acid sequence alignment of Egl from *Drosophila melanogaster* (fruit fly; NP_001286779.1), *Aedes aegypti* (yellow fever mosquito; XP_021697003.1), *Tribolium castaneum* (red flour beetle; XP_969046.1), *Apis mellifera* (honey bee; XP_026298567.1), and *Pediculus humanus* (human body louse; XP_002424820.1). Domains of Egl and intervening linker regions are indicated. Residues implicated in RNA base pair discrimination are labeled. Residues involved in RNA backbone interactions are marked with an asterisk (*), and residues contributing to inter-domain interactions are denoted with a circle. Conserved amino acids are colored according to the ClustalW coloring scheme: hydrophobic residues (A, V, L, I, M, F, W) are shown in blue; positively charged residues (K, R) in red; negatively charged residues (D, E) in magenta; polar residues (S, T, N, Q) in green; cysteine (C) in coral; glycine (G) in orange; proline (P) in yellow; histidine (H) in cyan; and tyrosine (Y) in turquoise.

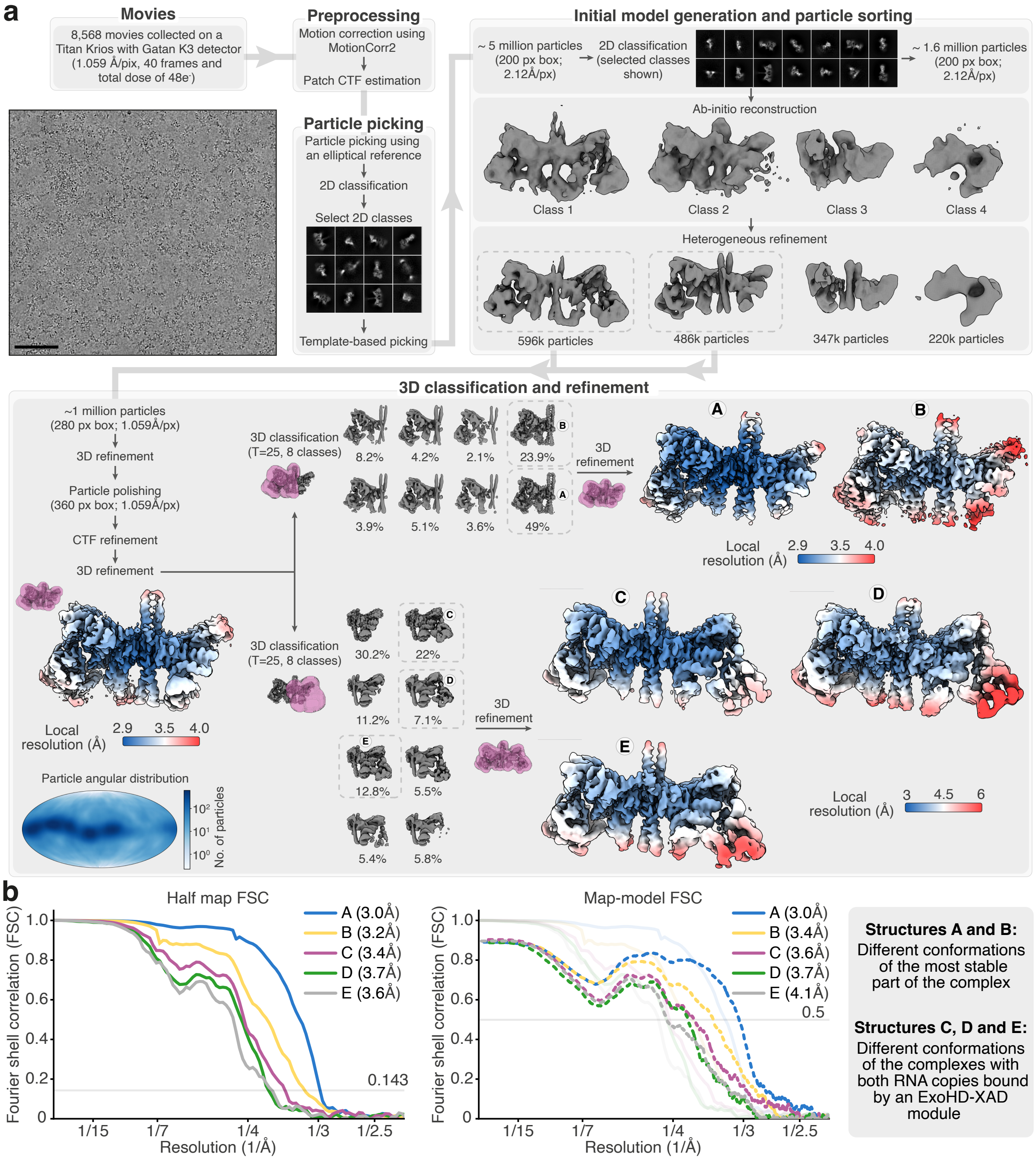

Supplementary Fig. 3 | Cryo-EM image processing pipeline for the Egl–BicD–*TLS* complex. a, Image processing was performed using cryoSPARC and RELION-4.0/5.0 and is described in detail in the Methods section. The classes selected after heterogeneous refinement and 3D classification are indicated with a dotted box. Unless otherwise specified, 3D classifications were performed without alignment (T = Tau fudge). The masks used for 3D classification and 3D refinements are shown in magenta. The angular distribution of particles, projected onto a Mollweide map, shows the orientation coverage of the particles used to obtain the consensus reconstruction. The consensus and final refined maps (structures A–E) are colored based on local resolution estimates from RELION. **b**, Fourier Shell Correlation (FSC) for structures A–E. The left panel shows the FSC between two independently refined half-maps, with resolution estimated using the 0.143 cutoff. The right panel displays the FSC between the final map and its corresponding model, with resolution estimated using the 0.5 cutoff.

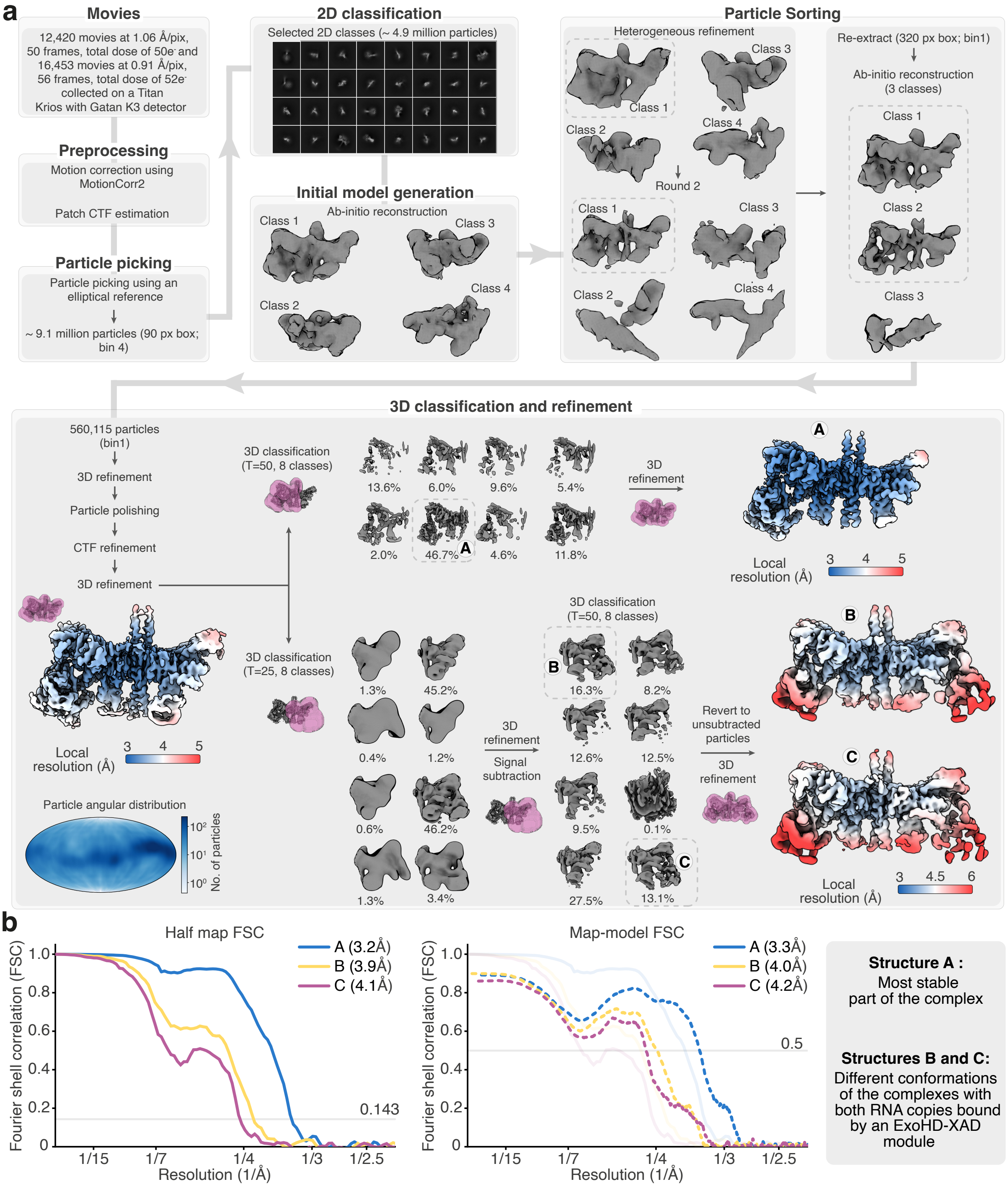

Supplementary Fig. 4 | Cryo-EM image processing pipeline for the Egl–BicD–*hSL1* complex. a, Image processing was performed using cryoSPARC and RELION-4.0/5.0 and is described in detail in the Methods section. The classes selected after heterogeneous refinement and 3D classification are indicated with a dotted box. Unless otherwise specified, 3D classifications were performed without alignment (T = Tau fudge). The masks used for 3D classification and 3D refinements are shown in magenta. The angular distribution of particles, projected onto a Mollweide map, shows the orientation coverage of the particles used to obtain the consensus reconstruction. The consensus and final refined maps (structures A–C) are colored based on local resolution estimates from RELION. **b**, Fourier Shell Correlation (FSC) for structures A–C. The left panel shows the FSC between two independently refined half-maps, with resolution estimated using the 0.143 cutoff. The right panel displays the FSC between the final map and its corresponding model, with resolution estimated using the 0.5 cutoff.

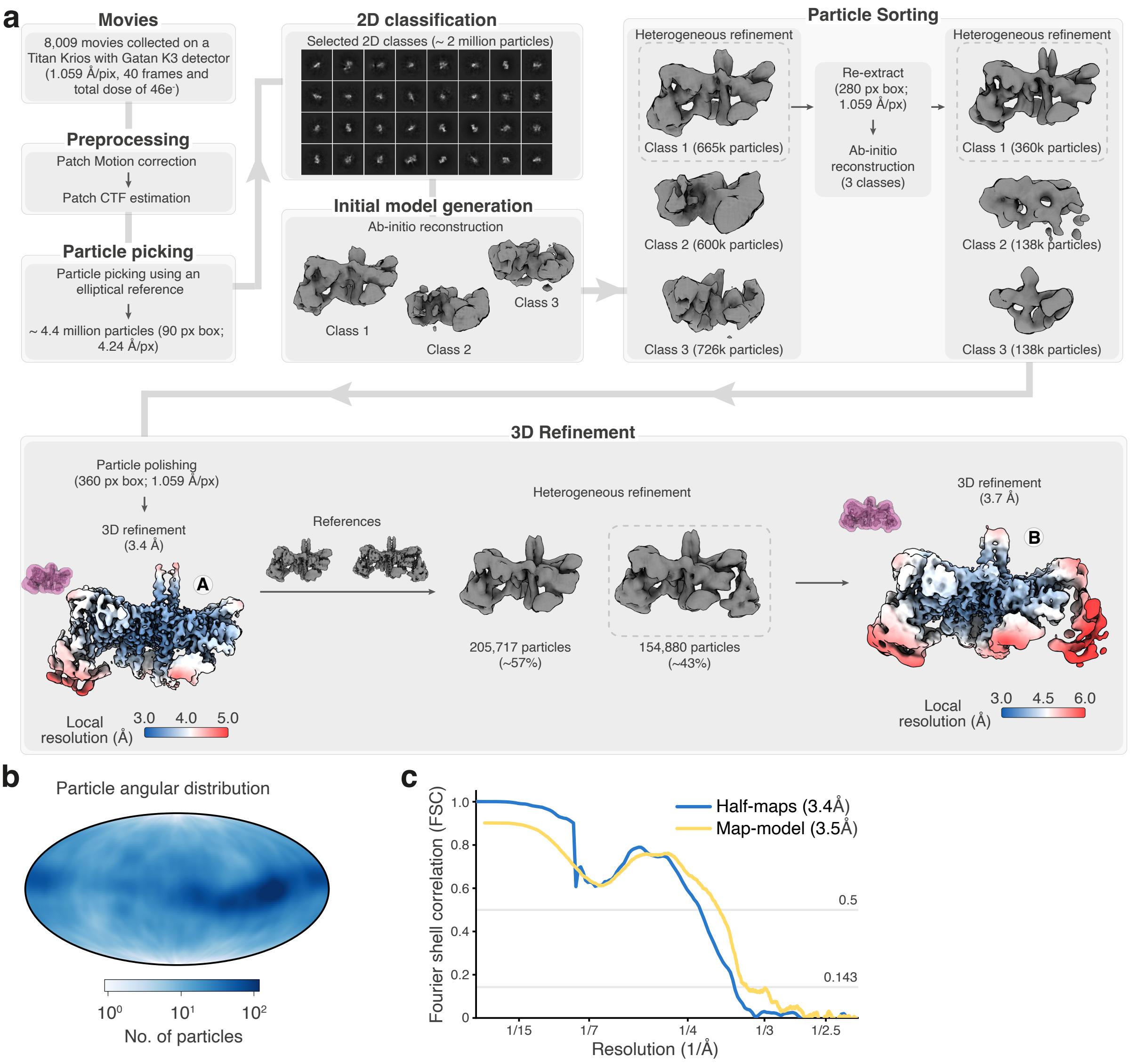

Supplementary Fig. 5 | Cryo-EM image processing pipeline for the Egl–BicD–*ILS* complex. a, Image processing was performed using cryoSPARC and RELION-4.0/5.0 and is described in detail in the Methods section. The classes selected after heterogeneous refinement are indicated with a dotted box. Masks used for 3D refinements are shown in magenta, and the final refined maps (structures A and B) are colored based on local resolution estimates from RELION. **b**, The angular distribution of particles, projected onto a Mollweide map, shows the orientation coverage of the particles used to obtain the consensus reconstruction (structure A). **c**, Fourier Shell Correlation (FSC) plot for structure A, showing the correlation between two independently refined half-maps, with resolution estimated using the 0.143 cutoff, and the correlation between the final map and its corresponding model, with resolution estimated using the 0.5 cutoff.

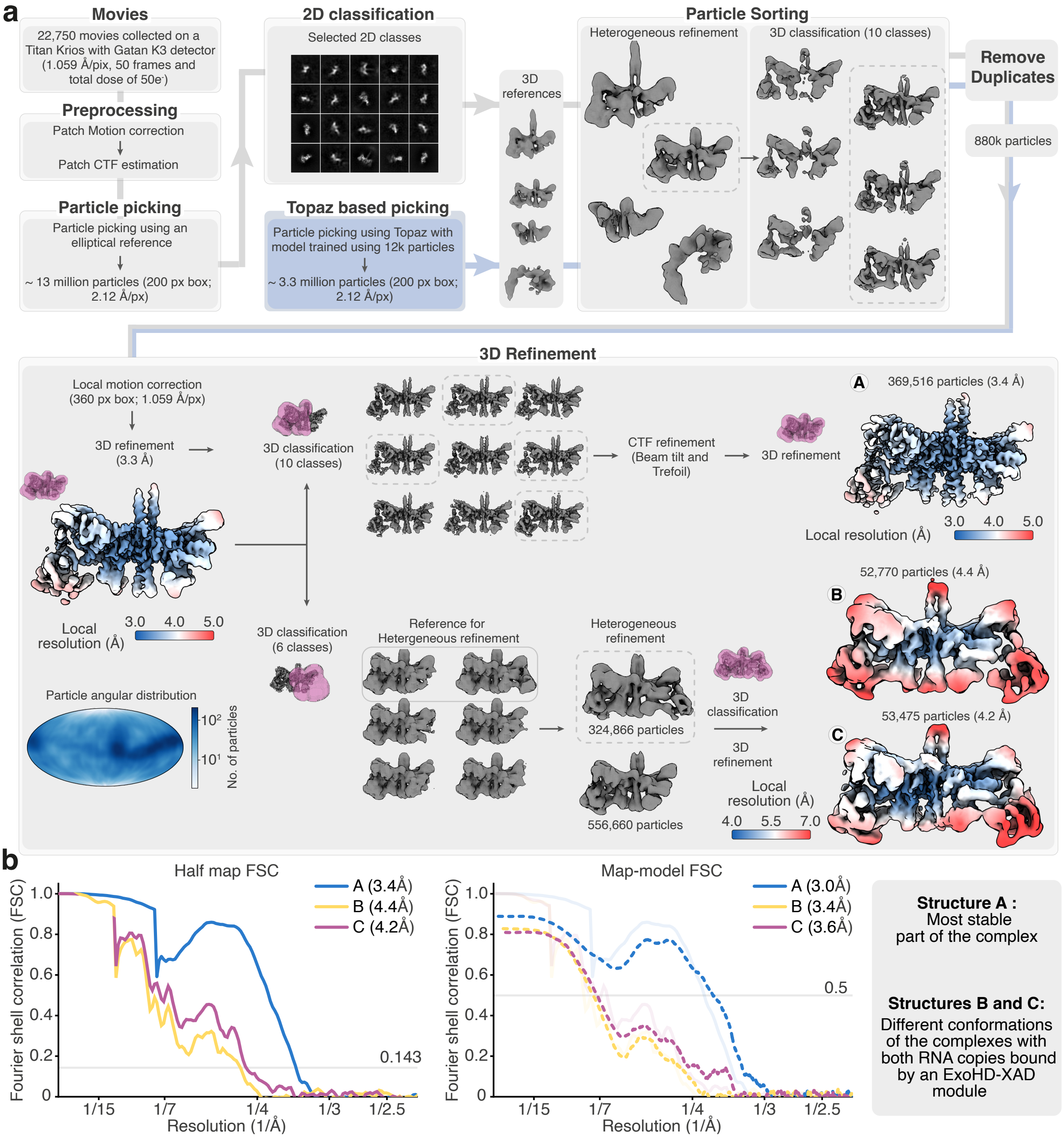

Supplementary Fig. 6 | Cryo-EM image processing pipeline for the Egl–BicD–*bcdSLV* complex. **a**, Image processing was performed using cryoSPARC and is described in detail in the Methods section. The classes selected after heterogeneous refinement and 3D classification are indicated with a dotted box. The masks used for 3D classification and 3D refinements are shown in magenta. The angular distribution of particles, projected onto a Mollweide map, shows the orientation coverage of the particles used to obtain the consensus reconstruction. The consensus and final refined maps (structures A–C) are colored based on local resolution estimates from RELION. **b**, Fourier Shell Correlation (FSC) for structures A–C. The left panel shows the FSC between two independently refined half-maps, with resolution estimated using the 0.143 cutoff. The right panel displays the FSC between the final map and its corresponding model, with resolution estimated using the 0.5 cutoff.

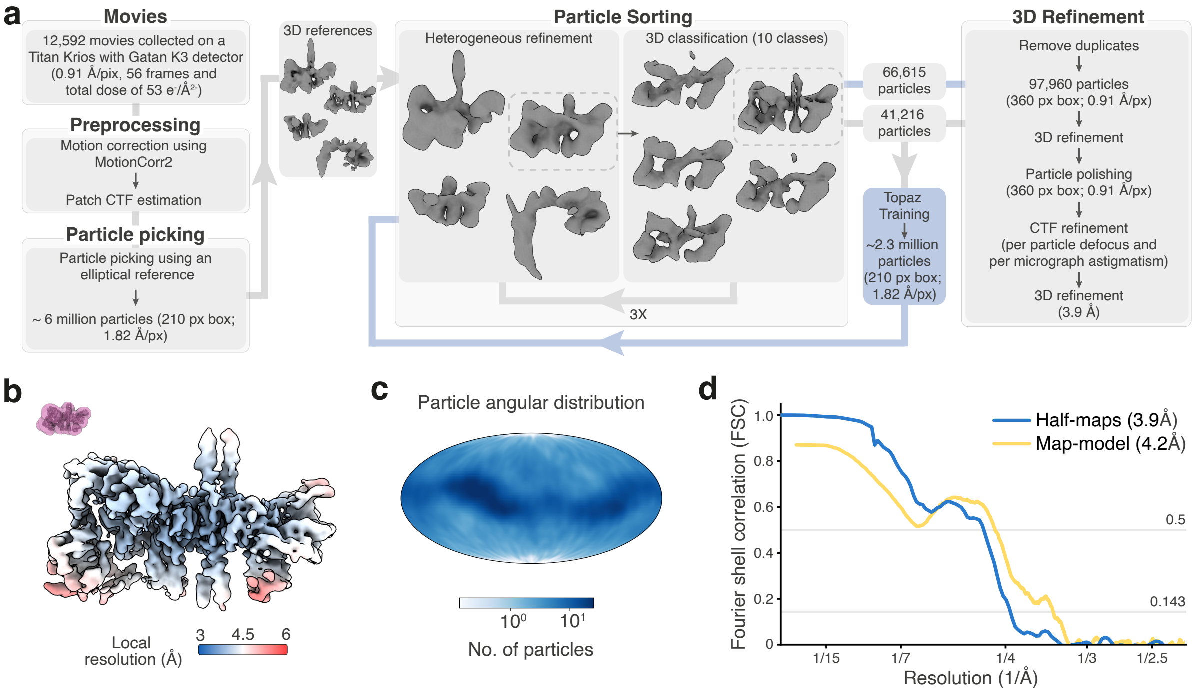

Supplementary Fig. 7 | Cryo-EM image processing pipeline for the Egl–BicD–*GLS* complex. a, Image processing was performed using cryoSPARC and RELION-4.0/5.0 and is described in detail in the Methods section. The classes selected after heterogeneous refinement and 3D classification are indicated with a dotted box. Unless otherwise specified, 3D classifications were performed without alignment. **b**, The final Egl–BicD–*GLS* structure colored based on local resolution estimates from RELION is depicted. The mask used for 3D classification and 3D refinements is shown in magenta. **c**, The angular distribution of particles used for the final reconstruction projected onto a Mollweide map is shown. **d**, Plot showing the FSC between two independently refined half-maps, with resolution estimated using the 0.143 cutoff, and the FSC between the final map and its corresponding model, with resolution estimated using the 0.5 cutoff.

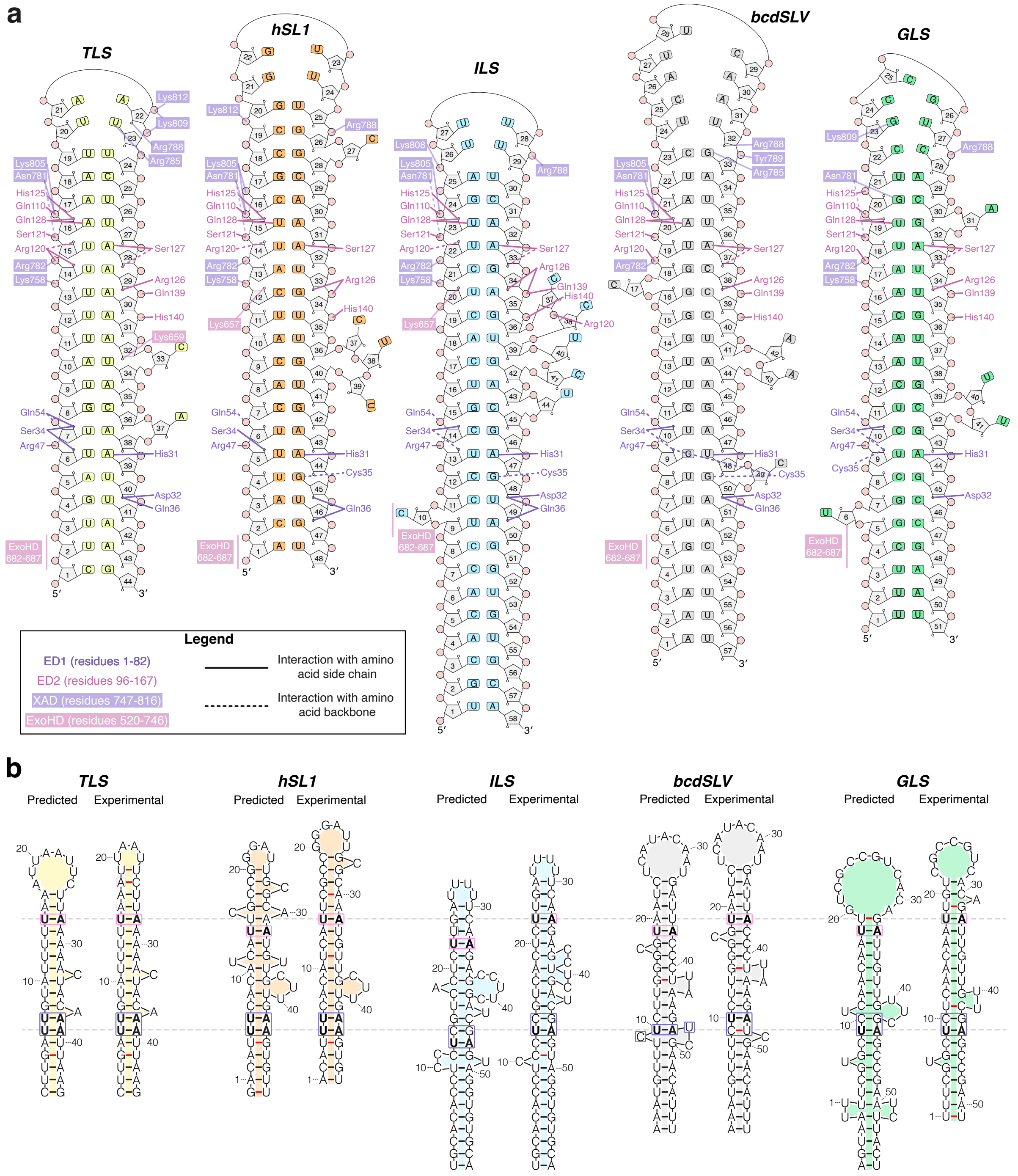

Supplementary Fig. 8 | Localization signal secondary structure and interactions with Egl. a, Secondary structure diagrams of the five RNA localization signals depicting the Egl contacts with bases and the ribose-phosphate backbone. RNA contacts made by Egl side chains are shown as solid lines and contacts made by the polypeptide backbone are shown as dashed lines. **b**, Comparison of predicted versus empirically determined secondary structures for the five localization signals. Predicted structures were generated by RNAfold whereas empirically determined structures were generated from cryo-EM data. Non-canonical base pairs are indicated by red lines. Base pairs adjacent to ED1 residues His31 and Cys35, and ED2 residue Ser127 are boxed, with the U–A base pairs at these positions shown in bold. Experimental stem loops are aligned to the base pair positions at which ED1 and ED2 make contact (denoted by dashed line).

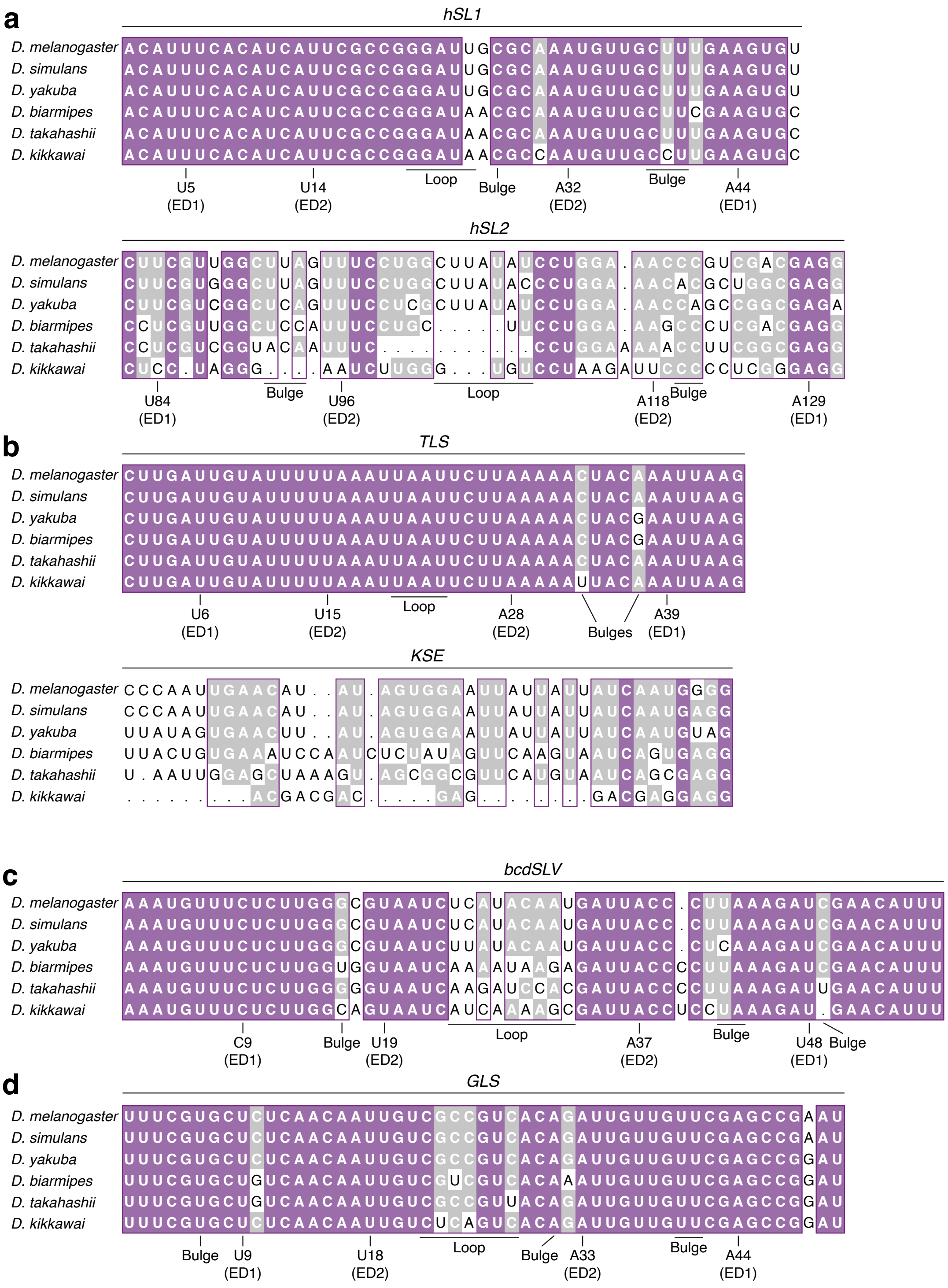

Supplementary Fig. 9 | Multiple sequence alignments of localization signals and support elements from divergent *Drosophila* species. a–d, Alignments of sequences of *TLS* and *KSE* (from *K10*) (**a**), *bcdSLV* (from *bicoid*) (**b**), *hSL1* and *hSL2* (from *hairy*) (**c**), and *GLS* (from *gurken*) (**d**) from indicated *Drosophila* species (approximate divergence times from *D. melanogaster*: *D. simulans*, 2.5 MA; *D. yakuba*, 5 MA; *D. biarmipes*, 13 MA; *D. takahashii*, 13 MA; *D. kikkawai*, 25 MA[1]). Alignments of the *ILS* are not shown as an active *I-factor* system appears to be restricted to the melanogaster subgroup and its close relatives[2]. Purple shading indicates complete conservation and gray shading indicates conservation amongst the majority of selected species at each position. For primary localization signals, extensive conservation outside of ED1 and ED2 binding sites suggests that these sequences optimize the stem-loop structure for Egl binding or associate with other RBPs that control different aspects of mRNA function.

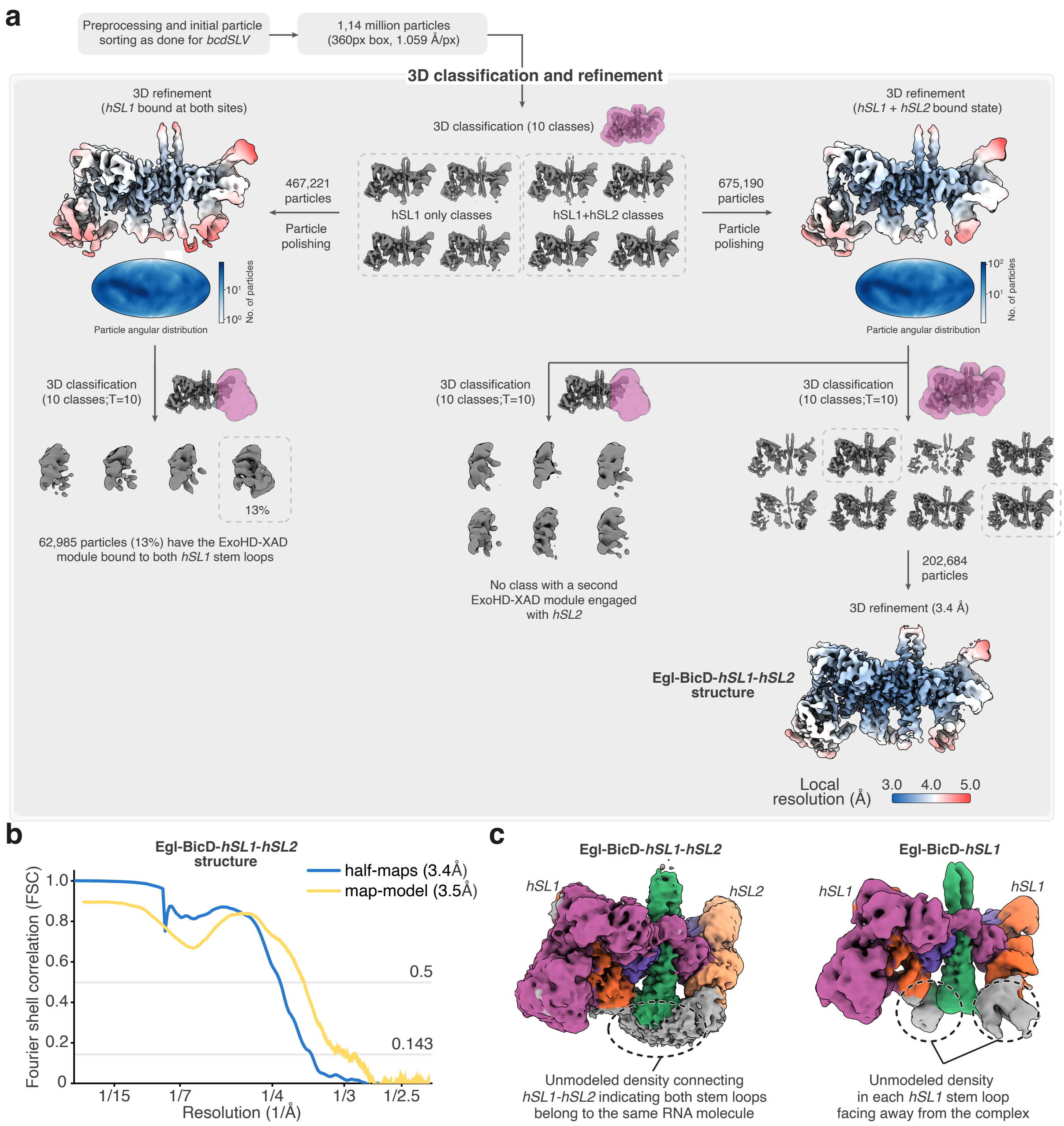

Supplementary Fig. 10 | Cryo-EM image processing pipeline for the Egl–BicD–*hSL1-hSL2* complex. a, Image processing was performed using cryoSPARC and RELION-5.0 and is described in detail in the Methods section. The classes selected after 3D classification are indicated with a dotted box. Unless otherwise specified, 3D classifications were performed without alignment (T = Tau fudge). The masks used for 3D classification and 3D refinements are shown in magenta. The angular distribution of particles, projected onto a Mollweide map, shows the orientation coverage of the particles used to obtain the consensus reconstruction. The consensus and final refined maps are colored based on local resolution estimates from RELION. **b**, Plot showing the FSC between two independently refined half-maps for the Egl–BicD–*hSL1-hSL2* complex, with resolution estimated using the 0.143 cutoff, and the FSC between the final map and its corresponding model, with resolution estimated using the 0.5 cutoff. **c**, Cryo-EM density maps of complexes formed in the presence of RNA containing both *hSL1* and *hSL2* (left) or *hSL1* alone (right). At a low-density threshold, the bases of the *hSL1* and *hSL2* stem loops are connected, showing they originate from the same RNA molecule.

hSL2

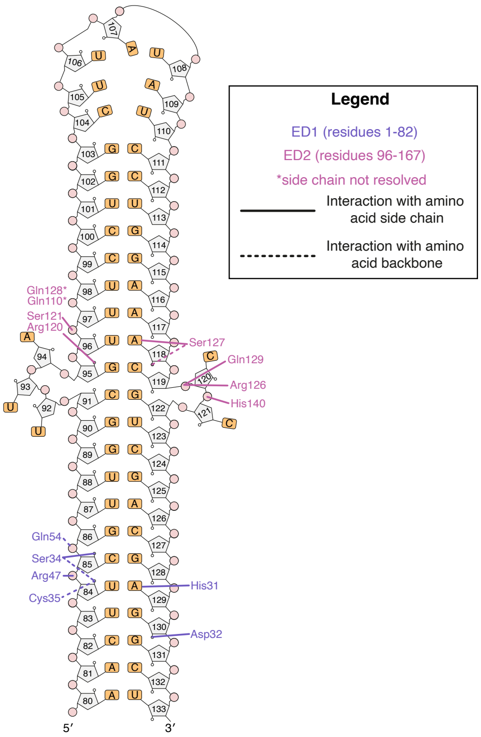

Supplementary Fig. 11 | Interactions of Egl with *hSL2*. Secondary structure diagram of *hSL2* depicting Egl contacts with bases and ribose-phosphate backbone. RNA contacts made by Egl side chains are shown as solid lines and contacts made by the polypeptide backbone are shown as dashed lines.

Supplementary Tables

Supplementary Table 1: Base pair inclination angles of localization signal stem loops as determined from their best-resolved structure obtained in this study.

Base pair	*PDB-2NUE*	*TLS*	*hSL1*	*ILS*	*bcdSLV*	*GLS*	*hSL2*
1	11.9	12.1	11.3	22.1	-1	20.1	5.6
2	12.2	13.9	5.8	23.4	4.5	13.5	7.8
3	14.4	11.8	14.5	19.5	9.1	9.6	6.9
4	11.9	13.2	9.8	20.2	9.6	9.5	12
5	12.8	11.1	5.5	19.5	7.9	5.8	13.6
6	14.9	7.2	7.7	9.1	8.8	-1	14.1
7	14.6	8.8	10.9	7.6	6.3	-0.8	12.5
8	12.7	8.3	11	4.4	2.9	4	14.4
9	13.8	11.7	9.3	-3.8	9.1	0.8	18
10	12	13.6	12	17.8	12.1	9.2	22.5
11	14.2	13.3	13.8	11.9	9.2	12.7	21.1
12	12.2	14.9	9.8	11	8.4	13.6	12.8
13	11.8	12.6	5.9	14.6	13.9	13.7	20.3
14	11.6	11.1	2.7	23.7	16.5	14.5	15.7
15	12.4	14.6	-1.3	14	14.7	11.7	11.3
16	9.6	14.8	62.6	20.1	20.9	11.5	10
17	11.2	11.9	1.8	-21.5	14	11.2	13.1
18	12.2	13.6	12.2	18.1	17.5		12.8
19	9.8		11.4	11.5	15.9		11.7
20	10.1			12.7	10.7		13.8
21	9.9			19.3	4.8		9.3
22				20.2	10.3		
23				23.2			

Base pair numbering starts at the base of each stem loop. Values were calculated using Curves+ and are presented in units of degrees (º). Values for the stem loop from PDB-2NUE are representative of A-form RNA.

Supplementary Table 2: Major groove widths of localization signal stem loops as determined from their best-resolved structure obtained in this study.

Base pair	*PDB-2NUE*	*TLS*	*hSL1*	*ILS*	*bcdSLV*	*GLS*	*hSL2*
1							
2							
3							
4							
5		5.9	10	5.3	6.6	7.1	6.4
6	4.7	6.2	8.3	5.6	6.9	9.2	5.2
7	4.8	6.3	5.3	5.6	5.7	10.7	5.5
8	4.4	4.8	5.1	4.4	6.4	7.5	5.8
9	3.9	3.6	4.5	5	7.2	6.5	5.7
10	4.6	4.5	4.9	5.9	7.3	5	6
11	5.5	5.3	4.7	7.3	3.8	4.7	8.4
12	5.9	5.7	6.2	7.2	3.4	5	9.5
13	6.5	6	6.5	5.3	4.8		11.7
14	6.3		5.9	6.8	5.6		15.3
15	5.1			5.6	5.5		9.3
16	5.1			4.3	5.4		5.7
17	5.9			5.2	5.2		
18				5.3	9		
19							
20							
21							
22							
23							

Base pair numbering starts at the base of each stem loop. Values were calculated using Curves+ and are presented in units of angstrom (Å). Values for the stem loop from PDB-2NUE are representative of A-form RNA.

Supplementary Table 3: Sequences of RNA stem-loop constructs used in this study.

Transcript	Sequence
TLS	UUACACCACUUGAUUGUAUUUUUAAAUUAAUUCUUAAAAACUACAAAUUAAGAUCACUCU
TLS$^{\Delta b}$	UUACACCACUUGAUUGUAUUUUUAAAUUAAUUCUUAAAAAUACAAUUAAGAUCACUCU
TLS-2xGC^{ED1}	UUACACCACUUGAGGGUAUUUUUAAAUUAAUUCUUAAAAACUACACCUUAAGAUCACUCU
TLS-GC^{ED2}	UUACACCACUUGAUUGUAUUUUGAAAUUAAUUCUUCAAAACUACAAAUUAAGAUCACUCU
TLS-2xGC^{ED1}*+GC*^{ED2}	UUACACCACUUGAGGGUAUUUUGAAAUUAAUUCUUCAAAACUACACCUUAAGAUCACUCU
TLS-GC$^{ED1/ED2}$	UUACACCACUUGAGUGUAUUUUGAAAUUAAUUCUUCAAAACUACAACUUAAGAUCACUCU
TLS-CG$^{ED1/ED2}$	UUACACCACUUGACUGUAUUUUCAAAUUAAUUCUUGAAAACUACAAGUUAAGAUCACUCU
TLS-AU$^{ED1/ED2}$	UUACACCACUUGAAUGUAUUUUAAAAUUAAUUCUUUAAAACUACAAUUUAAGAUCACUCU
KSE	AAACCCAAUUGAACAUAUAGUGGAAUUAUUAUUAUCAAUGGGGAAUAUUUAACCC
ILS	aaUGCACACCUCCCUCGUCACUCUUGAUUUUUCAAGAGCCUUCGAUCGAGUAGGUGUGCA
ILS$^{\Delta b}$	aaUGCACACCUCUCGUCACUCUUGAUUUUUCAAGAGUGACGAGAGGUGUGCA
hSL1	UGCGCGUAGACAUUUCACAUCAUUCGCCGGGAUUGCGCAAAUGUUGCUUUGAAGUGUUGCAAACAU
hSL1$^{\Delta b}$	UGCGCGUAGACAUUUCACAUCAUUCGCCGGGAUUGGCAAAUGUUGUGAAGUGUUGCAAACAU
hSL2	AACUUCGUUGGCUUAGUUUCCUGGCUUAUAUCCUGGAAACCCGUCGACGAGG
bcdSLV (EM)	AAAUGUUUCUCUUGGGCGUAAUCUCAUACAAUGAUUACCCUUAAAGAUCGAACAUUU
bcdSLV (MST)	aaAAAUGUUUCUCUUGGGCGUAAUCUCAUACAAUGAUUACCCUUAAAGAUCGAACAUUU
bcdSLV$^{\Delta b}$	aaAAAUGUUUCUCUUGGGGUAAUCUCAUACAAUGAUUACCCUAAGAUGAACAUUU
GLS (EM)	AGUAAUUUUCGUGCUCUCAACAAUUGUCGCCGUCACAGAUUGUUGUUCGAGCCGAAUCUUACU
GLS (MST)	aaAGUAAUUUUCGUGCUCUCAACAAUUGUCGCCGUCACAGAUUGUUGUUCGAGCCGAAUCUUACU
GLS$^{\Delta b}$	aaAGUAAUUUUCGGCUCUCAACAAUUGUCGCCGUCACAGAUUGUUGCGAGCCGAAUCUUACU
2NUE RNA	aaGGGACAAGCGCAAGGUCAUUCGCAAGAGUGGCCUUGCGCUUGUCCC
hSL1-hSL2	GCGCAUGCGCGUAGACAUUUCACAUCAUUCGCCGGGAUUGCGCAAAUGUUGCUUUGAAGUGUUGCAAACAUGCGAAUCCUAAACUCGGUUCACAACUUCGUUGGCUUAGUUUCCUGGCUUAUAUCCUGGAAACCCGUCGACGAGGCUAAGG
TLS-KSE	gucaaagcuuacCUUGAUUGUAUUUUUAAAUUAAUUCUUAAAAACUACAAAUUAAGuugcaaacaugcgaauccuaaacucgguucacaacuucCCCAAUUGAACAUAUAGUGGAAUUAUUAUUAUCAAUGGGGAAUAUgcuagc

Nucleotides in lower case represent sequences not present in the native transcripts.

Supplementary References

1. Ometto, L. et al,. Linking genomics and ecology to investigate the complex evolution of an invasive *Drosophila* pest. *Genome Biol. Evol.* 5, 745-757, doi.org/10.1093/gbe/evt034 (2013).
2. Bucheton, A., Simonelig, M., Vaury, C. & Crozatier., M. Sequences similar to the I transposable element involved in I-R hybrid dysgenesis in *D. melanogaster* occur in other *Drosophila* species. *Nature* 322, 650-653, doi.org/10.1038/322650A0 (1986).
