# Supplementary material for: Structural basis for recognition of diverse localizing mRNAs by Egl–BicD
Source: Nat Struct Mol Biol. 2026 May 5;33(5):882–93. doi: 10.1038/s41594-026-01794-8 (PMC13186709; doi:10.1038/s41594-026-01794-8)
Supplement: Supplementary file 2 — Reporting Summary [file 41594_2026_1794_MOESM2_ESM.pdf]

nature portfolio

Corresponding author(s): Mark McClintock, Andrew Carter and Simon Bullock

Last updated by author(s): Mar 20, 2026

Reporting Summary

Nature Portfolio wishes to improve the reproducibility of the work that we publish. This form provides structure for consistency and transparency in reporting. For further information on Nature Portfolio policies, see our Editorial Policies and the Editorial Policy Checklist.

Statistics

For all statistical analyses, confirm that the following items are present in the figure legend, table legend, main text, or Methods section.

n/a	Confirmed	
☐	☒	The exact sample size (*n*) for each experimental group/condition, given as a discrete number and unit of measurement
☐	☒	A statement on whether measurements were taken from distinct samples or whether the same sample was measured repeatedly
☐	☒	The statistical test(s) used AND whether they are one- or two-sided<br>*Only common tests should be described solely by name; describe more complex techniques in the Methods section.*
☒	☐	A description of all covariates tested
☐	☒	A description of any assumptions or corrections, such as tests of normality and adjustment for multiple comparisons
☐	☒	A full description of the statistical parameters including central tendency (e.g. means) or other basic estimates (e.g. regression coefficient) AND variation (e.g. standard deviation) or associated estimates of uncertainty (e.g. confidence intervals)
☐	☒	For null hypothesis testing, the test statistic (e.g. *F*, *t*, *r*) with confidence intervals, effect sizes, degrees of freedom and *P* value noted<br>*Give P values as exact values whenever suitable.*
☒	☐	For Bayesian analysis, information on the choice of priors and Markov chain Monte Carlo settings
☒	☐	For hierarchical and complex designs, identification of the appropriate level for tests and full reporting of outcomes
☒	☐	Estimates of effect sizes (e.g. Cohen's *d*, Pearson's *r*), indicating how they were calculated

Our web collection on statistics for biologists contains articles on many of the points above.

Software and code

Policy information about availability of computer code

Data collection	Zeiss ZEN 2.1 (confocal microscope image acquisition software-version 14.0.0.201); MicroManager (TIRF microscope image acquisition software version-1.4.22); MO.control (MST acquisition software-version 2.5.4); AlphaFold2; ThermoFisher EPU (CryoEM acquisition software-version 3.13)
Data analysis	FIJI v2.16.0/1.54p; RNAfold v2.6.3; mfold v3; Curves+ v2.6; ChimeraX v1.10; GraphPad Prism v10.4.0; ISOLDE v1.10.1; COOT v1.1.19; PHENIX v2.0; RELION (4.0 and 5.0); Topaz v0.2.5; CryoSPARC v5; MO.Affinity analysis v3.0.5. Custom code for analysis of RNA bending angles is available at https://github.com/carterlablmb/RNA_bend_analysis

For manuscripts utilizing custom algorithms or software that are central to the research but not yet described in published literature, software must be made available to editors and reviewers. We strongly encourage code deposition in a community repository (e.g. GitHub). See the Nature Portfolio guidelines for submitting code & software for further information.

Data

Policy information about availability of data

All manuscripts must include a data availability statement. This statement should provide the following information, where applicable:

- Accession codes, unique identifiers, or web links for publicly available datasets
- A description of any restrictions on data availability
- For clinical datasets or third party data, please ensure that the statement adheres to our policy

Data availability
Atomic coordinates and cryo-EM maps have been deposited in the Protein Data Bank (PDB) or Electron Microscopy Data Bank (EMDB), respectively, under accession codes 9RVY and 54292 (Egl–BicD–TLS structure A), 9RVZ and 54293 (Egl–BicD–TLS structure B), 9RW0 and 54294 (Egl–BicD–TLS structure C), 9RW1 and 54295 (Egl–BicD–TLS structure D), 9RW2 and 54296 (Egl–BicD–TLS structure E), 9RW3 and 54297 (Egl–BicD–hSL1 structure A), 9RW4 and 54298 (Egl–BicD–hSL1 structure B), 9RW5 and 54299 (Egl–BicD–hSL1 structure C), 9RW6 and 54300 (Egl–BicD–ILS structure A), 9RW7 and 54302 (Egl–BicD–bcdSLV structure A), 9RW8 and 54303 (Egl–BicD–bcdSLV structure B), 9RW9 and 54304 (Egl–BicD–bcdSLV structure C), 9RWA and 54305 (Egl–BicD–GLS), 9RWB and 54306 (Egl–BicD–hSL1–hSL2).
For Egl–BicD–ILS structure B, only the map was deposited in EMDB under the accession code 54301. Numerical source data for all plots are included with this paper.
All other data, including large imaging datasets, are available upon reasonable request to the corresponding author.

Research involving human participants, their data, or biological material

Policy information about studies with human participants or human data. See also policy information about sex, gender (identity/presentation), and sexual orientation and race, ethnicity and racism.

Reporting on sex and gender	Not applicable
Reporting on race, ethnicity, or other socially relevant groupings	Not applicable
Population characteristics	Not applicable
Recruitment	Not applicable
Ethics oversight	Not applicable

Note that full information on the approval of the study protocol must also be provided in the manuscript.

Field-specific reporting

Please select the one below that is the best fit for your research. If you are not sure, read the appropriate sections before making your selection.

☒ Life sciences ☐ Behavioural & social sciences ☐ Ecological, evolutionary & environmental sciences

For a reference copy of the document with all sections, see nature.com/documents/nr-reporting-summary-flat.pdf

Life sciences study design

All studies must disclose on these points even when the disclosure is negative.

Sample size	Sample sizes for Drosophila experiments were chosen based on the number of eggs that can be collected and processed in a cohort before aging beyond the blastoderm stage and precedents set in the literature, which capture reproducible differences in localization efficiency of RNAs. For single molecule data, the number of individual observations was determined by the number of microtubule-bound complexes per chamber, which were typically on the order of several hundred. These sample sizes are comparable to or larger than similar studies in the literature.
Data exclusions	Embryos that were clearly damaged at the injection site by the needle were not analyzed.
Replication	All results were reproducible in multiple replicates per experiment. RNA injections were replicated in 2 to 6 independent experiments. Pilot experiments showed that localization efficiency for a given RNA was consistent across different experiments. Single molecule observations were consistently made in 3 or 4 independent experiments. Affinity constants were determined by 3-6 independent MST measurements and no significant variability was observed in the affinities of different protein preparations for their RNA targets.
Randomization	Not applicable. All embryos from a given cohort were processed for injection with the same RNA.
Blinding	The experimenter performing injections was blind to the identity of the RNA being studied in order to eliminate unconscious bias

Reporting for specific materials, systems and methods

We require information from authors about some types of materials, experimental systems and methods used in many studies. Here, indicate whether each material, system or method listed is relevant to your study. If you are not sure if a list item applies to your research, read the appropriate section before selecting a response.

Materials & experimental systems

n/a	Involved in the study
☒	☐ Antibodies
☐	☒ Eukaryotic cell lines
☒	☐ Palaeontology and archaeology
☐	☒ Animals and other organisms
☒	☐ Clinical data
☒	☐ Dual use research of concern
☒	☐ Plants

Methods

n/a	Involved in the study
☒	☐ ChIP-seq
☒	☐ Flow cytometry
☒	☐ MRI-based neuroimaging

Eukaryotic cell lines

Policy information about cell lines and Sex and Gender in Research

Cell line source(s)	Sf9 (ECACC 89070101) derived from pupal ovarian tissue of the fall armyworm Spodoptera frugiperda. Commercially sourced from ThermoFisher Scientific
Authentication	Cell line was not authenticated by DNA analysis but had the stereotypical appearance of Sf9 cells
Mycoplasma contamination	Sf9 insect cells were routinely confirmed as Mycoplasma-free using the MycoALERT kit (Lonza).
Commonly misidentified lines (See ICLAC register)	None

Animals and other research organisms

Policy information about studies involving animals; ARRIVE guidelines recommended for reporting animal research, and Sex and Gender in Research

Laboratory animals	Drosophila melanogaster (w1118 strain)
Wild animals	Not applicable
Reporting on sex	The sex of Drosophila embryos cannot be determined at the age we studied. However, approximately equal numbers of male and female Drosophila embryos would have been injected with RNA in each experiment
Field-collected samples	Not applicable
Ethics oversight	No ethical approval was required for this work

Note that full information on the approval of the study protocol must also be provided in the manuscript.

Plants

Seed stocks	Not applicable - no plants used
Novel plant genotypes	Not applicable
Authentication	Not applicable
