# Supplementary figures and images for: Structural basis for recognition of diverse localizing mRNAs by Egl–BicD
Source: Nat Struct Mol Biol. 2026 May 5;33(5):882–93. doi: 10.1038/s41594-026-01794-8 (PMC13186709; doi:10.1038/s41594-026-01794-8)

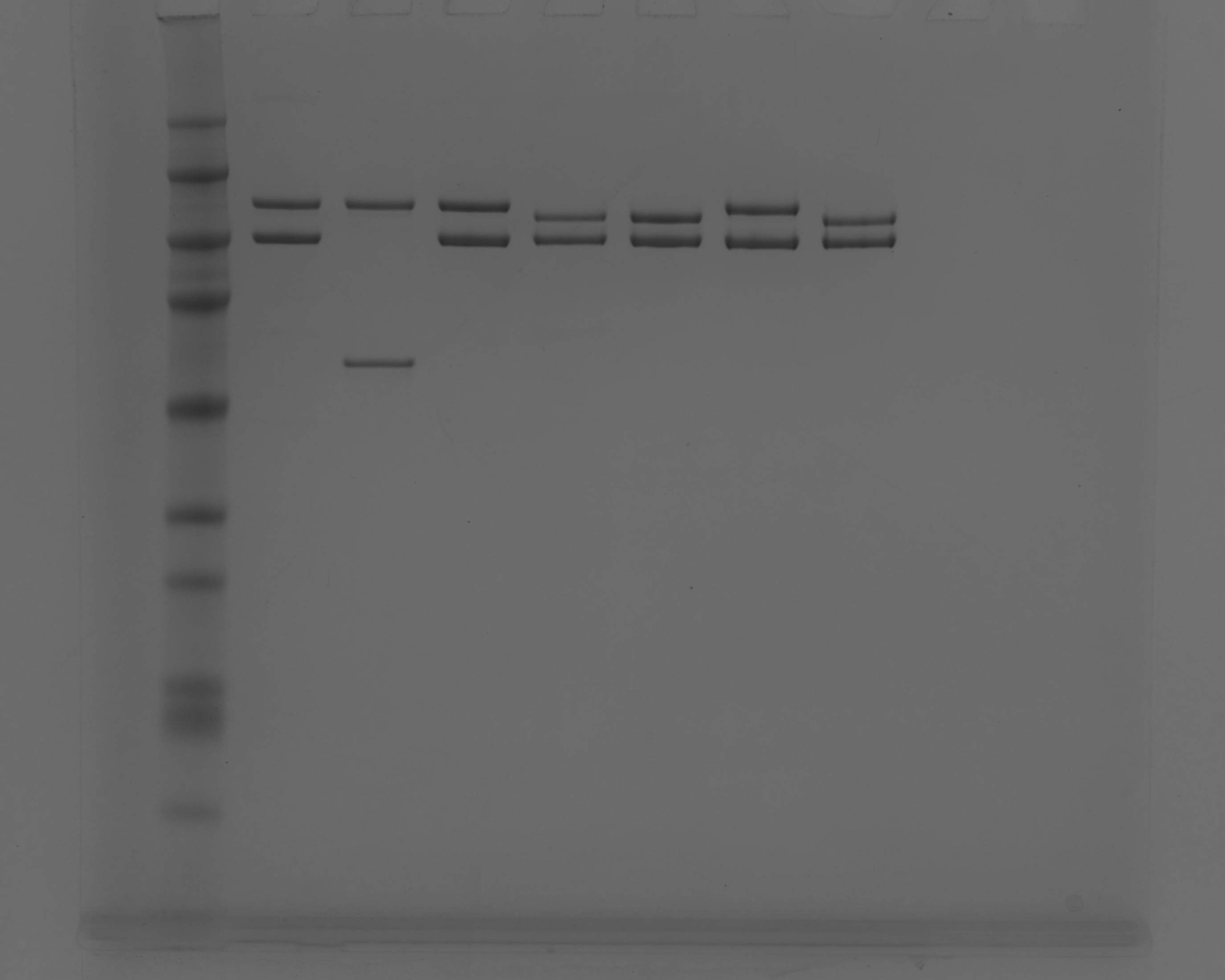

Supplement: Supplementary file 9 — Uncropped image of gel. [file 41594_2026_1794_MOESM9_ESM.tif]
